# Building biorepositories in the midst of a pandemic

**DOI:** 10.1017/cts.2021.6

**Published:** 2021-02-05

**Authors:** Jennifer A. Croker, Robin Patel, Kenneth S. Campbell, Marietta Barton-Baxter, Shannon Wallet, Gary S. Firestein, Robert P. Kimberly, Olivier Elemento

**Affiliations:** 1 Center for Clinical and Translational Science, University of Alabama at Birmingham, Birmingham, AL, USA; 2 Division of Clinical Microbiology, Division of Infectious Diseases, Mayo Clinic, Rochester, MN, USA; 3 Center for Clinical and Translational Science, University of Kentucky, Lexington, KY, USA; 4 North Carolina Translational and Clinical Sciences Institute, University of North Carolina, Chapel Hill, NC, USA; 5 Altman Clinical & Translational Science Institute, University of California San Diego, San Diego, CA, USA; 6 Clinical & Translational Science Center, Caryl and Israel Englander Institute for Precision Medicine, Weill Cornell Medicine, New York, NY, USA

**Keywords:** Biorepository, COVID-19, specimen, sample, CTSA, regulatory, SARS-CoV-2, biobanking IRB, informed consent

## Abstract

Biospecimen repositories play a vital role in enabling investigation of biologic mechanisms, identification of disease-related biomarkers, advances in diagnostic assays, recognition of microbial evolution, and characterization of new therapeutic targets for intervention. They rely on the complex integration of scientific need, regulatory oversight, quality control in collection, processing and tracking, and linkage to robust phenotype information. The COVID-19 pandemic amplified many of these considerations and illuminated new challenges, all while academic health centers were trying to adapt to unprecedented clinical demands and heightened research constraints not witnessed in over 100 years. The outbreak demanded rapid understanding of SARS-CoV-2 to develop diagnostics and therapeutics, prompting the immediate need for access to high quality, well-characterized COVID-19-associated biospecimens. We surveyed 60 Clinical and Translational Science Award (CTSA) hubs to better understand the strategies and barriers encountered in biobanking before and in response to the COVID-19 pandemic. Feedback revealed a major shift in biorepository model, specimen-acquisition and consent process from a combination of investigator-initiated and institutional protocols to an enterprise-serving strategy. CTSA hubs were well equipped to leverage established capacities and expertise to quickly respond to the scientific needs of this crisis through support of institutional approaches in biorepository management.

## Introduction

Since January 2020, the world has witnessed the grave societal impact of the newly emerged SARS-CoV-2 virus on morbidity and mortality, not to mention serious effects of the infection on national and global economies. Responses to COVID-19 were challenged to develop diagnostics and therapeutics for SARS-CoV-2 and to rapidly weave together current understanding of viral pathogenesis and novel insights of the impact of this virus on human physiology. Scientific investigation of COVID-19 demanded immediate organizational pivots considering the unique circumstances of a pandemic, the likes of which have not occurred in over a century. This mandate led to an immediate need for access to high quality, well-characterized COVID-19-associated biospecimens. Such access is essential for well-powered investigations of biologic mechanisms, validation of disease-associated biomarkers, development, validation and verification of diagnostics assays, recognition of microbial evolution and identification of targets for intervention that lead to development of new therapeutics [1].

The process of acquiring, processing, storing and distributing biospecimens from patients is complex. Specimen biorepositories have traditionally played a vital role in acquisition and management of disease-specific biospecimens. A variety of models and operational strategies have been used in the development of biorepositories. These range from collections to address specific, disease-oriented questions (project-driven), to those with a broader focus to address population-based goals to improve diagnosis and prevention of a wide variety of health conditions (general). Models may be centralized (single coordinated collection, standardized operating procedures), federated (multiple collection points, standardized procedures) or decentralized (individualized approaches, variable procedures), or hybrid, that is, a combination of these approaches [2]. Important considerations for development of a biorepository include the consenting process, frequency and timing of collections (cross-sectional, longitudinal), specimen-types and volumes involved, quality assurance for sample processing, appropriate facilities to protect against biohazards that may be present in the specimens, linkage to clinical phenotypic information and rigorous management tactics for tracking storage, retrieval and distribution. A biorepository must consider ethical, legal, and regulatory factors in the downstream use of specimens and sharing of data [3]. A prioritization strategy is needed to utilize specimens in studies that offer the greatest scientific impact.

The need for robust repositories became immediately apparent as COVID-19 hit academic medical centers. Broadly, all non-essential research was shut-down or slowed while COVID-19 research was authorized. Special considerations regarding COVID-19 biorepositories led to rapid evaluation of a diversity of biorepository approaches across institutions. Pre-COVID-19 pandemic biorepositories faced numerous challenges, ranging from limited consent, high costs of banking and sample annotation, alignment between collected specimens and investigator needs, unequal sample utilization, and processes for prioritization and governance. The COVID-19 pandemic highlighted these challenges, exacerbated some of them and added new ones as well. For example, acute sample governance and utilization issues have arisen as a result of COVID-19 specimens being in limited supply and COVID-19 samples only being processed in elevated biosafety level (BSL) environments (e.g., BSL2-enhanced laboratories or, for viral cultivation, BSL3 facilities). Experiences during the pandemic provide an opportunity to learn from diverse approaches across institutions, enabling improvements in routine day-to-day functions and preparation for future pandemics. To better understand the nature and impact of the pandemic influence on biorepositories, we surveyed biomedical research centers across the country about COVID-19 biorepository practices. The objective of the survey was to gather information about biorepository practices prior to COVID-19 and to understand how practices were altered and redefined as a result of the pandemic. From the results of this survey, we highlight key lessons learned, durable changes and describe opportunities to address future global health challenges.

## Results

Representatives of Clinical and Translational Science Award (CTSA) hubs – a national network of medical research institutions sponsored by the National Institutes of Health to accelerate the translation of scientific discoveries to improve patient health (https://ncats.nih.gov/ctsa/about/hubs) – were invited to participate in a survey to describe strategies and barriers encountered in biobanking before and in response to the COVID-19 outbreak. The survey was administered by the Center for Leading Innovation & Collaboration (CLIC) Survey Team in October, 2020. Data were collected via REDCap from a total of 60 institutions representing all regions of the continental United States. A total of nine biorepository-related questions were posed, relying on a combination of multiple choice and free-text response formats, with brief descriptions of concepts to provide context (Appendix 1). All questions offered an open-ended feedback option to provide additional information. The survey was limited by the need for conciseness as CTSAs were also asked to provide input on a range of other topics not directly related to biorepositories.

### Biorepository Models

**Fig. 1.**
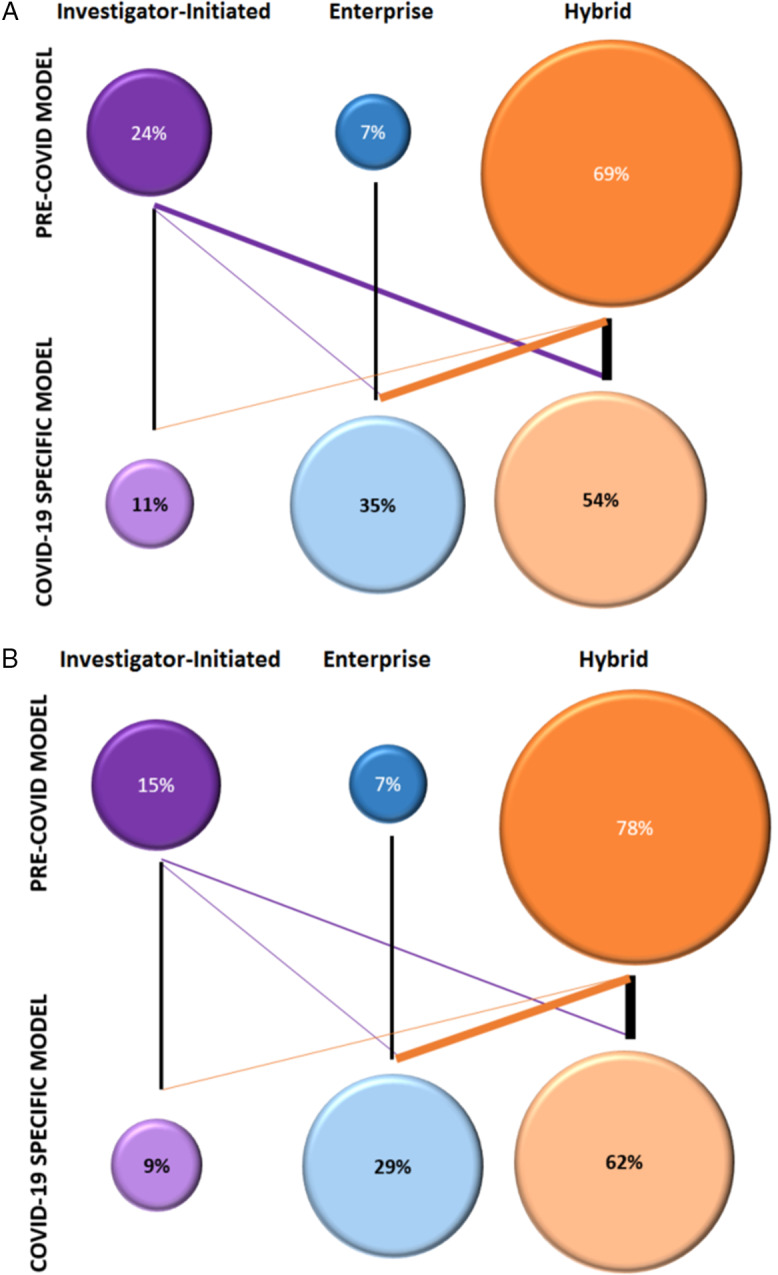
Shifts in Biorepository Approaches before (pre-COVID-19) and in specific response to COVID-19. A. Comparison of the approaches in biorepository models (investigator-initiated, enterprise, hybrid). B. Observed shifts in participant consent and specimen acquisition. (Percentages reflect fraction of total survey respondents; weight of lines represents relative proportion of the shift).

Before COVID-19, a majority (69%) of institutions applied a hybrid model of biorepository management, involving both coordinated and investigator-initiated strategies, with an individual approach used in 24%, and an enterprise-wide, institutional approach being uncommon (7%) (Fig. 1A). As a result of the pandemic, a dramatic shift in biorepository models was observed, to a more prominent enterprise approach (35%), with 54% using a hybrid approach and a small number (11%) retaining individually-oriented efforts.

A similar trend was observed at a more granular level in the approach to participant consent procedures and specimen acquisition. Pre-COVID-19 recruitment and collection procedures adopted a combination of investigator-specific and institutionally-driven approaches (78%). In response to the pandemic, enterprise-based recruitment and specimen acquisition increased nearly four-fold, from 7% to 29% (Fig. 1B).

**Fig. 2.**
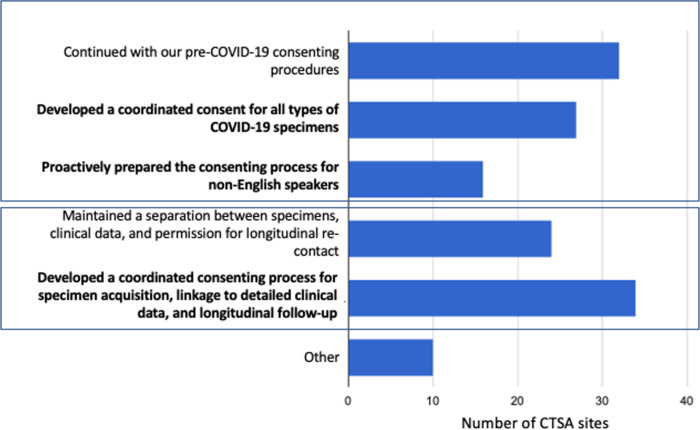
Changes made to biospecimen acquisition and banking consenting procedures due to COVID-19. CTSA, Clinical and Translational Science Awards.

We specifically asked CTSAs what changes were made to biospecimen acquisition and banking consenting procedures due to COVID-19. Approximately half (27 of 59; 46%) of the institutions surveyed developed a coordinated consent for collecting COVID-19 specimens, with 27% (16 of 59) implementing processes and translated documents for non-English speakers (e.g., Spanish). A majority (34 of 59; 58%) went further to develop a coordinated consenting process for specimen acquisition, linkage to detailed clinical data, and longitudinal follow-up. The remainder maintained distinct separation between specimens, phenotypic information and permissions for re-contact (Fig. 2).

We also asked questions related to specimen governance, utilization, and funding (Table 1). The launch and management of biorepositories during the health crisis prompted widespread adoption of coordinated governance structures (73%), with nearly half of the surveyed institutions also establishing scientific advisory committees to address research prioritization. While these committees and institutions mostly permitted use for academic collaborations (86%) and consortia (66%), a meaningful subset (24%) made specimens and data available to industry collaborators. Establishment, growth and ongoing maintenance of COVID-19 biorepositories relied on a variety of financial resources, including investigator grants and recharge, center core grants and specialized cooperative agreements (e.g., CTSA), institutional commitments from Schools of Medicine, Health Systems and/or other units, and philanthropy.

**Table 1.** Primary approach for COVID-19 specimen governance, prioritized use, and financial support

	COVID-19-specific [(%), # CTSA sites]
**Specimen Oversight**	
Creation of a governance group, which has guided the acquisition of specimens and helped to prioritize sample allocation	(73%), 43
Created a scientific advisory group to anticipate specimen needs and obtain them proactively (e.g., pediatric samples, health care workers, in-patient, out-patient, non-patient research participants)	(46%), 27
**Allowed Specimen Use by:**	
Academic Collaborators	(86%), 51
Academic Consortia	(66%), 39
Industry Collaborators	(24%), 14
**Sponsorship**	
Investigator Grants or charge	(73%), 43
Center Grants and Mechanisms (e.g., CTSA)	(69%), 41
Funds from the School of Medicine, Health System or other Schools	(68%), 40
Philanthropy	(47%), 28

CTSA, Clinical and Translational Science Awards.

**Fig. 3.**
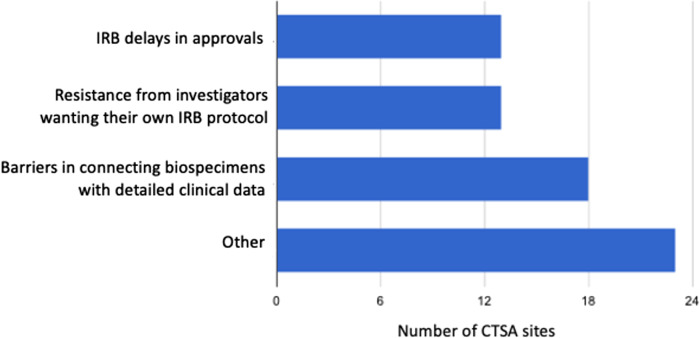
Major obstacles institutions encountered in enabling biorepository activities to support COVID-19 related research. “Other” category includes issues such as lack of funding, overwhelming demand, absence of biosafety level capacity, access to patients, and staffing challenges. CTSA, Clinical and Translational Science Award; IRB, Institutional Review Board.

Finally, we requested that CTSAs provide input on obstacles that institutions encountered in enabling biorepository activities to support COVID-19 related research (Fig. 3). The survey results indicated a number of obstacles, including delays in human subjects regulatory approvals (41 of 47; 28%), resistance from investigators wanting their own IRB protocols (13 of 47; 28%) and barriers in linking specimens to detailed clinical information (18 of 47; 38%).

## Discussion, Lessons Learned and Perspectives for the Future

Traditionally, approaches to support biospecimen repositories have been diverse in structure and approach. A common model for biorepositories has involved investigator-initiated, disease-oriented, prospective collections. Programs supported by the National Institutes of Health, including NCATS-sponsored Clinical and Translational Science Award (CTSA) hubs and NCI-designated Comprehensive Cancer Centers, have played a significant leadership role in promoting coordinated and organized biorepository management, including oversight and quality control/assurance. Often these resources rely on sophisticated tracking software, including OnCore biospecimen management (BSM), OpenSpecimen, Velos eSample or customized REDCap systems. The linkage to clinical data, manually abstracted from medical records, obtained directly from EHR, or augmented by patient surveys, is critical and adds tremendous value to the specimens. In some cases, annotated samples are digitally accessible or integrated into informatics platforms (e.g., i2b2) that allow specimen discovery based on clinical criteria.

### Immediate Challenges Encountered

The survey and our own experience showed that multiple factors unique to SARS-CoV-2 strained traditional strategies for human subjects research and biospecimen collection. In mid-March, as the World Health Organization formally declared the pandemic [4], the majority of academic health centers suspended or slowed non-COVID-19 research activities and in-person instruction. Non-essential workers were expected to stay home and telecommute, when possible. At the same time, clinical care facilities became increasingly stressed in managing severely infected individuals, a situation further compromised by supply chain limitations on personal protective equipment (PPE) [5]. Guided by recommendations authored by the Center for Medicare and Medicaid Services and Centers for Disease Control and Prevention [6, 7], many hospitals enacted COVID-19-related policies that restricted patient accompaniment or visitation in healthcare facilities in an effort to reduce the spread of infection.

As a result of these immediate changes, many CTSA sites anticipated and observed similar shifts in research needs that further complicated a stressed environment; many investigators were inspired to apply their expertise to better understand biologic mechanisms of SAR-CoV-2, despite limited funding for new areas of research. One of the most important consequences was a dramatic rise in number of human subject research protocols and efforts targeting a finite number of COVID-19 patients. Offices of Institutional Review Boards (IRBs) were swiftly overwhelmed at the same time their staffs were shifting to a virtual support arrangement.

Many challenges emerged (Box 1). Early on, individual researchers attempted to engage front-line clinical care staff to facilitate participant enrollment and specimen collection, at times frustrating those already overwhelmed with patient care responsibilities. At some sites, a few investigators strongly objected to an institutional approach and continued to approach clinicians and patients. Collection and consenting was hampered by evolving understanding of infectious risks and a limited supply of PPE. Frequently, specimen needs of different investigators overlapped, and experimental goals (e.g., cell, cytokine and antibody assays; host or viral genotyping) were redundant. This was exacerbated by the formation of numerous multi-institution consortia, with which investigators desired to share samples and data. Research objectives also quickly shifted as our collective understanding of the virus’ biology and its impact on the host evolved (for example, focus has recently evolved to include understanding long-term consequences post-infection and viral variants). In addition, specimen collection, without careful oversight, put participants at risk by possibly exceeding IRB allowances (e.g., multiple blood draws for research).

Box 1. Immediate Challenges

Patient experience and perspective

Incomplete and evolving understanding of risk of exposure to infected subjects (to obtain consent and collect specimens)

Incomplete and evolving understanding of risk of exposure to specimens (collecting, processing and storing specimens)

Consenting in non-traditional locations (e.g., drive-through settings)

Diversity of specimen-types available and needed

Specimen stability (e.g., viral RNA, peripheral blood mononuclear cells)

Evolving understanding of ideal specimen-types, and emergence of specimen types “new” to biobanking (e.g., saliva)

Study prioritization

Specimen allocation

Standardization of procedures

Adaptability (sample handing)

Linkage to clinical data

Supply chain shortages (ongoing)

Individual investigators approaching patients and clinicians, creating “biosample fatigue”

Early on, it was unclear exactly which specimen-types should be considered infectious, with uncertainty about how to handle blood, urine, stool, and other bodily fluids. Even once that uncertainty was clarified through scientific studies, many specimen-types, such as nasopharyngeal (NP) swabs, mid-turbinate swabs, anterior nares swabs, saliva, sputum, endotracheal aspirates, and bronchoalveolar lavage fluid continued to be recognized as being potentially hazardous, proving a challenge to ensure the safety of everyone involved in the workflow. Facilities’ needs (e.g., installation of biological safety cabinets), additional collection materials (e.g., swabs and transport media, many of which were and continue to be in short supply), testing reagents (many of which continue to be in short supply), and a shortage of qualified microbiology laboratory workers, further strained the system. Some institutions re-trained staff and deployed them to handle biobanking of COVID-19 specimens.

In addition to research purpose-collected specimens, many institutions developed processes to harness remnant specimens originally collected for clinical evaluation and diagnostic purposes, the latter needing a separate work flow from specimens collected exclusively for research purposes. The demands needed for specimen management strained, and continue to strain, laboratory medicine and pathology units that simultaneously needed to significantly ramp up COVID-19 diagnostic testing while faced with unprecedented supply chain shortages, requiring implementation and offering of multiple new platforms in many academic laboratories. For example, in August 2020 a majority of academic medical center and community hospital/health system laboratories reported using three or more SARS-CoV-2 testing methods in their clinical practices [8]. These differences also created challenges for research as specimens were not characterized by single standardized tests.

### The CTSA Hubs’ Response to Specimen Needs

As the urgent scientific need encountered a chaotic pandemic environment, academic health centers were immediately challenged with determining whether and how to support COVID-19 research. Our survey shows that many CTSA hubs took active steps to coordinate an institutional biorepository response that incentivized collaboration and data sharing. Such an approach sought to be broadly applicable and efficient. It also prioritized good stewardship and trusted engagement of research subjects who were affected by a poorly-understood virus and who were facing an unpredictable situation potentially involving severe morbidity and mortality. The complexity of their personal decision-making process vis-à-vis participation in research obligated a coordinated approach.

Our survey and our own experiences showed that the foundation for this coordinated approach frequently relied on a specialized COVID-19 biorepository-focused human subjects (IRB) protocol that maintained participant protections while affording maximal flexibility in specimen use. Some protocols leveraged verbal consent strategies to minimize exposures and accepted consent from the participant, legal representatives (in cases of intubation) or next of kin (for autopsy). Some COVID-19 biorepositories obtained remnant specimens from clinical pathology units after completion of clinical testing (e.g., NP swabs). Biorepository efforts typically relied upon defined partnerships with clinical care teams to streamline specimen collections and assured workflows that reflected rigorous biosafety standards.

A majority of sites (58%) developed a coordinated consenting process for specimen acquisition, linkage to detailed clinical data, and longitudinal follow-up. In some instances, waivers of HIPAA authorization were obtained to link specimens with clinical phenotypic information via electronic health records (EHR) and to re-contact convalescent patients to gather information not available in the EHR. The reasoning behind such waiver involved the balance of the benefits of clinical COVID-19 research to better understand the pandemic with the risks to patients which were perceived to be minimal. Moreover, the centralized nature of biorepositories enabled linkage between samples and clinical data by a small number of well-trained, authorized individuals, while the large majority of users were provided access to de-identified data. Thus biorepository protocols were designed to enable as many research teams as possible, the latter having access to de-identified specimens for secondary analysis, often in the context of separate investigator-initiated, IRB-exempt projects. This strategy minimized risk of viral exposure across research teams and served to accelerate discovery by allowing studies to launch more quickly by leveraging an existing consented cohort and scientific resource. Furthermore, as the need for additional specimen types evolved with an increasing knowledge base, amendment of IRB protocols allowed biorepositories to adapt and be nimble in response to emerging scientific understanding. The urgency of the pandemic limited the harmonization of protocols and collection strategies across institutions.

With the shift in the biorepository practice to a coordinated model to support COVID-19 research, many common factors were observed across sites. Coordinated COVID-19 biorepositories often benefitted from expertise and capacity previously established by CTSA hubs. These units could take advantage of existing BSL2 laboratories that were enhanced to allow for processing of COVID-19 specimens, high-throughput laboratory information management systems (LIMS) and robotic handling and processing workstations. Where needed, additional facilities were upgraded to support BSL2+ or BSL3 standards to handle infectious materials in a variety of specimen types (Table 2). Specimen collections reflected institutional research strengths (e.g., immunology, microbiome, genetics/genomics, proteomics) in an effort to align resources with scientific needs of the most investigators. The ability to prescribe timing and frequency of collections was variable across sites due to the complexity of the clinical care environment and the dynamic nature of the infection. Some COVID-19 biorepositories also operationalized collection of convalescent samples in an effort to support longitudinal follow-up and to advance the understanding of long term sequelae. In some circumstances, protocols were able to operationalize special, investigator-specific requests. Coordinated biorepositories also benefited from involvement of expert microbiologists in laboratory medicine and pathology to understand and manage the unique specimen-types, characterization and handling associated with COVID-19.

**Table 2.** COVID-19 biospecimen collection strategy to meet research opportunities

Specimen Type	Experimental Use
Nasopharyngeal swabs, mid-turbinate swabs, anterior nares swabs, saliva, endotracheal aspirates, sputum, bronchoalveolar lavage fluid	Development, validation and verification of diagnostic tests (e.g., RT-PCR, Loop-mediated isothermal amplification (LAMP), antigen detection) and various specimen-types (to ease supply chain shortages, minimize risk to healthcare providers and increase patient satisfaction), studies of viral kinetics over time, viral evolution studies (i.e., through sequencing), infectiousness/contagiousness, host response markers, microbiome studies, etc.
Blood	Array-based genotyping, whole genome sequencing, exome sequencing
PBMC	Immunophenotyping, CyTOF (mass cytometry), single cell RNAseq, live cell populations, antibody repertoire
Serum/plasma	Proteomics, metabolomics, antibody, coagulation studies
Platelets	Coagulation studies
Autopsy	Multiplex imaging (imaging mass cytometry)
Fecal swabs, skin	Microbiome

PBMC, peripheral blood mononuclear cell.

The value of the specimen is directly related to the depth and breadth of phenotype information that can be provided. Enterprise COVID-19 biorepositories, in many cases, were able to leverage established digital ecosystems to link specimens to clinical data. Access to patient-reported outcomes, case report forms, and other data not systematically captured for clinical care were more difficult to support unless the institution already had methods and available staff in place to implement such collection.

Our survey revealed that, in many cases, a committee of multiple stakeholders was organized to provide transparency and fair access, a fundamental tenet of the enterprise-serving model. These governance committees would meet regularly (sometimes same-day) to adjudicate specimen requests relative to study design, scientific impact and specimen availability. They were also utilized to prioritize processing approaches based on an assessment of research community demand, processing cost, personnel time and scientific value (e.g., preparation of peripheral blood mononuclear cells, T cells, B cells, single cell sorting). The committee also assessed sharing specimens outside of the organization (e.g., with academic collaborators, scientific consortia, industry partners). This governance committee, or a complementary scientific advisory team, helped to identify important targeted populations (e.g., pediatrics, healthcare workers, inpatient/outpatient, non-patient research participants) to anticipate specimen needs and to work through related logistics and regulatory concerns to support such collections, especially in the case of vulnerable groups. A challenge associated with the governance structure has been the need to prioritize different studies and stay within reasonable guidelines. It is difficult, especially within organizations more accustomed to individual investigator-driven structures, to balance individual expectations with efforts to enable multiple investigators, team science and the community of scholarship.

### Lessons Learned

COVID-19 biorepository approaches developed during the first six months of the coronavirus pandemic have developed traction in recruiting patients and collecting, processing, storing and distributing a variety of specimens to support a broad range of scientific investigation. We found that overall, Clinical and Translational Science Award (CTSA) hubs were well equipped to adapt as needed and leverage established capacities and expertise to quickly respond to the scientific needs of this crisis. Nonetheless, several key lessons have been identified.

The importance of institutional biorepositories has been underscored. For a variety of reasons ranging from priorities and business models to governance challenges, institutional biorepositories have frequently not been funded at a level needed to support patient-oriented research, especially in the context of a pandemic. The institutional biorepository model can help overcome several of the obstacles that were revealed by the survey. For example, delays in human subjects regulatory approvals may be minimized if investigators can get access to samples via the institutional biorepository, and do not need to “reinvent the wheel” when it comes to both consent and sample processing in a resource restricted environment. Barriers in linking specimens to detailed clinical information can also be lowered when well vetted institutional procedures for such linking are already in place. The survey overwhelmingly revealed that CTSA hubs expect to see more resources allocated to institutional biorepositories in the future.

A second lesson identified has been the need for a variety of specimens from diverse populations of patients and controls to support high-quality clinical and translational research. To support rigorous, reproducible study design, biorepositories need to explore ways to recruit patients from populations most affected by the pandemic, as well as from well-defined patients under investigation and healthy controls. Informatics teams are working to curate concepts, like comorbid conditions and other variables that may not reflect unique variables, to further refine phenotype. Mindful of the significant resources – both financial and human capital – needed to build biorepositories, institutions need to continue to explore ways to increase enrollment success by taking advantage of video-based or e-Consent strategies, with special consideration of how such platforms may be received by patients across the lifespan. Biorepositories will also beneficially develop harmonized study protocols, clinical data and sample handling workflows to allow rigorous, multi-site investigation.

One obstacle that was noted by some CTSA hubs was resistance of some investigators wanting their own IRB protocols and collections. In some cases, well-intended efforts to lead a coordinated strategy, sensitive to patient perspective and guidelines for specimen collection, were misconstrued as a means to control the direction of research and constrain academic freedom. In the process of implementing a coordinated approach, COVID-19 biorepositories and institutional leaders need to enhance communication with investigators to better articulate the value proposition in the collaborative nature of utilizing biospecimen collections. The development of a specimen repository typically reflects an investment of intellectual and technical expertise, and access to appropriate samples relies on engaged and scholarly discussion to pair the optimal specimen to the design of the study. This value and investigator benefit was notably recognized in environments that shifted from a predominantly investigator-focused model of specimen collection pre-COVID-19 to a coordinated approach in response to the pandemic. The urgency of the pandemic, including considerations of patient perspective and safety as well as of safety for research team members, typically facilitated such coordination.

In many ways, it can be seen that COVID-19 has served as a catalyst for changes that were already underway (e.g., shift to e-consent). Institutions are encouraged to maintain and build on these gains to continue team-based processes that are sustainable and long-lasting (Box 2). Many of these changes will also help address potential global health challenges that may present in the future, including other pandemics. Nonetheless, additional measures may be needed. For example, federal rapid-response grants would greatly facilitate rapid deployment of resources for biobanking in case of global health crises and ensure that more high-quality biospecimens are collected for research. Institutions may better prepare in advance by fostering trusting relationships among investigative and clinical care teams and by developing a shared “play book” for facilitating research specimen collections in the context of a crisis. Increased incentives for biospecimen sharing across CTSA hubs may enable high-impact research that can achieve scale and statistical power (e.g., COVNET: Large-scale Genome-wide Association Study and Whole Genome Sequencing of COVID-19 Severity) and can more rapidly benefit patients. This may be a natural extension of existing sharing efforts among CTSAs such as the National COVID Cohort Collaborative (N3C) [9].

Box 2. Sustainable Commitments

eConsent/eSignature

Linkages to clinical data, informatics-enabled solutions

Translated consent documents (multi-lingual)

Rapid mobilization of resources for biobanking

Enterprise coordination, governance, scientific advisory input

Culture change towards institutional biobank efforts

Appropriate biosafety level capacity for biobanking

An increased focus on infectious diseases as a priority for biobanking

In summary, this national survey of CTSA hubs has identified important considerations for specimen repositories in biomedical research, especially in the midst of a crisis. Additional investigation is needed to characterize institutional and investigative team approaches to further enhance participant recruitment and biospecimen collections. This may include studies to understand resourcing, tools to increase diverse representation (e.g., multi-language consent), infrastructure requirements (e.g., freezers, data management, biosafety protocols) and inter-institutional policies to facilitate specimen discovery and collaborative use.

## Supplementary material

For supplementary material accompanying this paper visit http://dx.doi.org/10.1017/cts.2021.6. click here to view supplementary material
