# Supplementary material for: Building biorepositories in the midst of a pandemic
Source: J Clin Transl Sci. 2021 Feb 5;5(1):e92. doi: 10.1017/cts.2021.6 (PMC8134891; doi:10.1017/cts.2021.6)
Supplement: Supplementary file 1 [file S2059866121000066sup001.docx]

**APPENDIX 1 (Supplementary Material)**

***Special Communication***

**Building Biorepositories in the Midst of a Pandemic**

Jennifer A. Croker^1^, Robin Patel^2^, Kenneth S. Campbell^3^, Marietta Barton-Baxter^3^, Shannon Wallet^4^, Gary S. Firestein^5^, Robert P. Kimberly^1^, and Olivier Elemento^6^.

The following survey was conducted by the Center for Leading Innovation & Collaboration (CLIC) Survey Team in the Fall, 2020. This is an abridged version of the data collection instrument focusing on general demographic questions and Section 2: Biorepositories, the data from which were used to inform this manuscript.

**JCTS COVID-19 Special Edition Survey**

Each Hub PI and Hub administrator has received a unique REDCap link to the survey below. We ask that only one survey per hub is submitted by the Hub PI (with the assistance of the Hub administrator) via the online REDCap survey platform. Please submit the survey through the PI link unless the PI delegates the responsibility to the administrator, in which case use the administrator's link. If you are NOT the communicating PI, you should contact them for instructions. DO NOT forward or share either unique links with anyone else. The purpose of this survey is to solicit information on the creative innovations and adaptations made by the hubs in response to the COVID-19 pandemic. Findings will be submitted for publications in a special issue of the Journal of Clinical and Translational Science.

This survey covers eight different topics and you may need to solicit responses from others at your hub in order for you to complete the survey. To gather expert-specific data from colleagues in your hub, a Microsoft Word version was provided for internal use. [https://rochester.box.com/s/3e0jo3ytn4j32v2s87cdlkjswae3xesv] Key personnel are asked to complete the Word Document version survey and return it to the hub PI to complete the official online survey (e.g., sharing the Word version with several hub colleagues as needed, into which they can input data and information and return it to the PI/administrator). If you would like to send in multiple responses for any particular section, please send the word document with the multiple responses to survey@ctsa-clic.org AFTER the REDCap survey is submitted for your Hub. CLIC will forward those sections to the appropriate Writing Groups. You may also contact the Writing Group leads directly using the emails previously supplied to you.

Each topic will be found on its own page in the following order:

Section 1: N3C and Informatics

Section 2: Biorepositories

Section 3: Virtual Visits

Section 4: IRB

Section 5: Protecting Research Personnel

Section 6: Prioritizing COVID-19 Studies

Section 7: COVID-19 Adaptations to Pharmacy Procedures

Section 8: Prioritizing non-COVID-19 Studies

You will have the ability to save and return to the survey at any time using your unique survey link found in your email invitation. When you submit the survey, you will receive an email confirmation with a PDF of your response. We ask that you save your response for your records and for reference in case we need to reach out with any follow-up questions.

We would appreciate your collating all of the responses and submitting the REDCap version as quickly as possible, but no later than 11:59 PM, on Thursday, October 15, 2020. If you have any questions please contact [survey@ctsa-clic.org](mailto:survey@ctsa-clic.org)

Thank you,

CLIC Survey Team

**Hub Information**

Hub Name (Drop Down)

Please indicate the period of time in which you began to experience COVID-19 related disruptions to your research operations.

- Prior to January 2020
- January-March 2020
- April-June 2020
- July-September 2020
- NA
- Other

**Section 2: Biorepositories**

Which of the following best describes your biorepository model (Pre-COVID-19 and COVID-19 Specific)

(Please only select one model per column)

|  | Pre-COVID-19 | COVID-19 Specific |
| --- | --- | --- |
| Individual, investigator-initiated protocols and consents |  |  |
| Enterprise approach for some but not all types of specimens (eg, remnant surgical tissue) |  |  |
| Enterprise approach with standard SOPs for all biospecimens |  |  |
| Hybrid Approach |  |  |
| Other |  |  |

Which of the following best describes your institution's biospecimen acquisition and banking consenting

procedures? (Pre-COVID-19 and COVID-19 Specific) Check all that apply.

|  | Pre-COVID-19 | COVID-19 Specific |
| --- | --- | --- |
| Individual, investigator-initiated protocols and consents |  |  |
| Enterprise approach for some but not all types of specimens (eg, remnant surgical tissue) |  |  |
| Enterprise approach with standard SOPs for all biospecimens |  |  |
| Hybrid Approach |  |  |
| Other |  |  |

Which of the following describe changes made to your biospecimen acquisition and banking consenting procedures due to COVID-19? Check all that apply.

- Continued with our pre-COVID-19 consenting procedures
- Developed a coordinated consent for all types of COVID-19 specimens
- Proactively prepared the consenting process for non-English speakers
- Maintained a separation between specimens, clinical data, and permission for longitudinal re-contact
- Developed a coordinated consenting process for specimen acquisition, linkage to detailed clinical data, and longitudinal follow-up
- Other

Did your institution develop special governance to help manage acquisition and allocation of COVID-19 related biospecimens? Check all that apply.

- Continued with our individual, investigator-initiated approach
- Created a governance group, which has guided the acquisition of investigator-requested samples and helped to prioritize sample allocation
- In addition to governance, we created a scientific advisory group to anticipate specimen needs and obtain them proactively
- Other

Which of the following group interests were prioritized when developing a governance and prioritization process to help balance internal vs. external requests for COVID-19 biospecimens? Check all that apply.

- Academic colleagues
- Academic consortia
- Industry collaborators
- Other

How did your institution support expanded demands for your biorepository activities to accommodate COVID-19 related research? Check all that apply.

- Individual investigator grants and charges
- Center grants and mechanisms, like the CTSA
- Funds from the School of Medicine, Health System, other schools
- Philanthropy
- Other

What were the major obstacles that your institution encountered in enabling biorepository activities to support COVID-19 related research? Check all that apply.

- IRB delays in approvals
- Resistance from investigators wanting their own IRB protocol
- Barriers in connecting biospecimens with detailed clinical data
- Other

What do you anticipate will be the long term impact of COVID-19 on biorepositories at your institution?

(Please consider the following) Do you anticipate that more resources will be allocated to biobanking than before? Will there be expanded use of e- or video-consent? Will more BSL2+ lab space be made available to biorepositories? Will there be changes in governance process? Do you expect any negative impact? Other?

Please provide any additional comments.

(Please consider the following) What recruitment strategies were more effective in the COVID-19 context? What recruitment strategies were less effective in the COVID-19 context? What consent strategies were more effective in the COVID-19 context? What consent strategies were less effective in the COVID-19 context? Did you encounter patient-specific considerations (e.g., special populations)?
